# Dr. D. Domingo Madan*It is a great pleasure to be able to present the life of so noble a Cuban physician.

**Published:** 1898-10

**Authors:** 


### DR. D. DOMINGO MADAN.*

Translated from *Crônica Médico-Quirurgica de la Habana*

BY B. J. WORD,

ATLANTA, GA.

Here is a name born to the life of science together with the *Crônica Médico-Quirurgica de la Habana,* in which he was a collaborator when yet a very young man. The inscrutable designs of nature have not permitted him to outlive this actual unfortunate epoch; he has fallen in the middle of his course; and this publication, always so dear to him, in which he leaves so many treasures of learning, and which at a more or less distant time he would have been called to manage, being one of the youngest of its first editors, devotes to him a tear and tries to-day, although not according to the full measure of its wishes, to present him to his countrymen, and also to posterity, with the triple encomium of altruist, patriot, and an exemplary physician. His calling for medicine and his generous and noble sentiments manifested themselves at an early period, when he was performing the functions of an assistant in the clinical wards for eye diseases of Dr. J. Santos Fernandez. Many are those upon whom he operated who remember yet the gentleness of that young man—young in years but mature in wisdom—when advising and persuading them; but when he established himself in his native town, Matanzas, the kindness of his heart was not yet developed, although it increased day after day and reached a height where his generosity and love towards his neighbor gave him the character of an apostle. In that provincial capital he loved so much, and which has claimed his remains, there is hardly a beneficent institution where his hand has not intervened, especially the Orphans', the Old People's Homes, and the Dispensary for Children, the first of its kind founded in Cuba, which have been his exclusive work. For three decades to the present day the beautiful city of Matanzas, justly called the "Athens of Cuba," has been the victim for many reasons of a decadence great and profound,

*It is a great pleasure to be able to present the life of so noble a Cuban physician.

which in these later days has become a real disaster, and there is no need to try to describe the effect which such had produced upon the sensitive soul of our friend. In vain he strove to counteract the effects of hunger and misery in the unhappy children, and with a solicitude never equaled before he attended them in the dispensary established by himself before the coming of this terrible situation. How he was infusing into his colleagues that same spirit of kindness which was flooding from him so profusely, so that the grand work of charity should indeed show result and be a living image of that of the Savior when he exclaimed, Let children come unto me—*Sinite parvulos venire ad me!*

For the aid of that dispensary, whose sustenance was chiefly the origin of the disease from which he died, Dr. Madan made a trip to Europe in order to study in a perfect manner this class of establishments for destitute children; but this was not the only time he crossed the seas for humanity's sake, as he went twice to Paris, appointed by the Bacteriological Laboratory of Havana, with the object of removing difficulties relative to the installation in Cuba of the prophylactic method for the treatment of hydrophobia conceived by Pasteur. And all this was willingly and cheerfully done, just as if there was no sacrifice at all to give up his interests and family, without any personal benefit whatever and only for the public welfare. If we would relate all the numerous episodes attached to these general points of his devotedness to good works, we would need many pages, but the reader will not miss those details although we do not write them, as they are a necessary consequence of an abnegation revealed in the most salient lines of his eminently altruistic character.

As a public man he never shirked any responsibility. He occupied in the municipality and other corporations the posts assigned to him by the vote of his fellow men. He always remained affiliated to the autonomist party and by the side of those who proclaimed the liberal ideas as a sure means of avoiding the explosion of impatiences born from the doubt of obtaining redress of inveterate wrongs from the supreme powers. He constantly advocated that concord so much in conformity with his temperament, and if he had political adversaries he never had very bitter ones, because his tolerance on one side and his conciliatory manners on the other permitted him to get from both something good for his country—the only object of his solicitude and cares.

When the clear sky of the island was darkened by the smoke of battles, when the report of cannons went through the air and resounded in his wounded soul, his complexion lost its former freshness, smiles fled from his lips, and his languid look revealed a constant mourning. All those who descended to the grave in that horrible struggle took with them in the folds of their mortuary clothes a breath of his life, and this repeated affliction, together with the one producing hunger and misery as an inevitable consequence of war, undermined his constitution, impaired by a fatigue provoked through his desire to heal them, and thus favored the triumph of the disease which carried him to the grave in spite of science and of the efforts of his colleagues and friends, who fought it eagerly in the hope of the return to health of a man so dearly respected.

He served his country as a member of the Boards of Public Instruction and Health, in which he worked assiduously; contributed to the maintenance of this publication, not only with the fruit of his deep intelligence, but also with a ready and seasonable advice. He understood that the "country" begins in the family, and he was a model of sons in the home where now lie overwhelmed with grief his virtuous parents, yesterday so happy, and so unfortunate in the present hour.

As soon as the people of Matanzas were made aware of the sad news of his death in Havana, at 4 o'clock P.M. on the 24th of July, they expressed their vehement desire to become the possessors of his remains. On the following day the whole city was waiting for them, and in the midst of tears of gratitude they were carried to the necropolis, where they are resting by the side of those three illustrious physicians, Presas, Llarac and Jiminez, for whom Madan professed so much respect and contributed in a great measure to the homage paid them, and which in a higher degree he receives himself from his native city as a just premium for his acknowledged patriotism.

His public spirit and the attention given to the calls of society, of which he was a prominent member in spite of his natural modesty, never prevented him from the practice of medicine and from his studies; and although there is a tendency to believe that only one can be obtained at a time, but never both together, Madan was writing what he was observing daily, and in this manner was creating our own medicine. In this lies the principal merit of his numerous memoirs, some published in the columns of this publication, others read before the society for clinical studies, and not a few published by the Royal Academy of Sciences, of which he was only a correspondent, on account of not being a resident of Havana. His first works were on ophthalmology, having collected all those of his master and publishing them in two volumes under the heading of "Clinica de enfermedades de los ojos." Later, he invaded courageously the field of pathology, and with great predilection for pediatrics. Close to a patient's bedside and in the presence of a rebellious disease, he rose giantlike and displayed with a skill above imitation the resources of a therapeutics which his fervent enthusiasm was expanding and studying in its effects. His great erudition was only revealed when in private and in a friendly conversation his advice was sought for. He was noted for two qualities seldom found together—a memory to utilize opportunely the ideas of others, and a privileged intelligence to discourse with effect and produce something new and original, by which he was placed at the head of our most conspicuous physicians.

---

### A MIXTURE FOR THE FETID DIARRHEA OF THE INITIAL STAGE OF SCARLATINA.

Filatov (*Revue mensuelle des maladies de l'enfance*, July) recommends the following;

| | | |
|---|---|---|
| ℞ | Sulphite of magnesium, Liquid sulphuric acid, } of each . . . . . . . . . . | 60 grains. |
| | Distilled water . . . . . . . . . . . . . . . . . . . . | 6 ounces. |
| | Syrup . . . . . . . . . . . . . . . . . . . . . . . . | 450 grains. |
| M. | | |

A teaspoonful or tablespoonful, according to the child's age, from hour to hour. This draught is markedly anodyne and is well taken by little children.

